# Association between cold spells and chronic lung disease: a nationwide spatial machine learning analysis

**DOI:** 10.3389/fmed.2026.1823044

**Published:** 2026-05-13

**Authors:** Xing-Ru Chen, Chun-Tao Liu

**Affiliations:** Department of Pulmonary and Critical Care Medicine, West China Hospital, Sichuan University, Chengdu, China

**Keywords:** CHARLS, chronic lung disease, cold spell, geographical Gaussian process regression, middle-aged and elderly

## Abstract

**Background:**

To investigate the longitudinal association between baseline cold spells and the risk of incident chronic lung disease (CLD) among middle-aged and elderly adults in China, and to characterize its spatial distribution and multifactorial context.

**Methods:**

Using national data from the China Health and Retirement Longitudinal Study (CHARLS, 2011–2020), Cox proportional hazards models, logistic regression, stratified analysis and sensitivity analyses were employed to analyze the impact of nine differently defined cold spell indicators on the risk of new-onset CLD in adults aged ≥45 years. Geographically Gaussian process regression (GGPR) mapped spatial disease distribution, and GeoShapley decomposition quantified factor contributions.

**Results:**

In Cox models, baseline cold spell exposure was associated with a modestly elevated risk of incident CLD (fully adjusted hazard ratios [HRs] ranged from 1.10 to 1.13). This positive association was further supported by logistic regression analyses (fully adjusted odds ratios [ORs]: 1.11–1.13) and sensitivity analyses. After correction for multiple comparisons, urban residence, higher education, and cancer history modified the association. Spatial prediction models (GGPR) identified northern China as a consistent high-risk region across all nine cold spell definitions (validation AUC: 0.7077–0.7129). Notably, GeoShapley analysis revealed that air pollutants and geographic coordinates contributed more substantially to spatial prediction than did cold spell exposure, indicating that regional background factors dominate the spatial patterning of CLD risk.

**Conclusion:**

Higher baseline cold spell exposure was associated with a modestly increased risk of incident CLD among Chinese adults aged ≥45 years, with northern China identified as a consistent high-risk region. The independent longitudinal association of cold spells, though not dominant in spatial prediction models, highlights their potential role as an environmental trigger warranting further investigation.

## 1 Introduction

Chronic lung disease (CLD), which encompass chronic bronchitis, emphysema, chronic obstructive pulmonary disease (COPD), and related obstructive airway disorders, constitute a major global cause of mortality and impose a substantial health and economic burden (1–5). Despite decreasing age-standardized rates, the absolute counts of CLD incidence, prevalence, mortality, and disability-adjusted life years (DALYs) are rising globally due to demographic shifts (6, 7). COPD alone is responsible for 84.27% of CLD deaths and 73.53% of DALYs, underscoring its dominant role (7). China is disproportionately affected, home to an estimated 100 million COPD patients, representing about one-quarter of the world’s cases (1). The aging population intensifies this burden, with older individuals being more vulnerable to cumulative risk factors such as smoking (8, 9). Notably, CLD burden escalates with age and is most severe in individuals aged 60 years and older (10, 11). Therefore, the elderly in China emerge as the highest-priority group for CLD management, calling for enhanced clinical attention and resource investment.

Beyond well-established risks such as tobacco smoke and occupational particulates (12), air pollution has emerged as a significant and modifiable environmental factor in CLD development. For instance, each 10 μg/m^3^ rise in PM₂.₅ exposure was associated with a 15% increase in chronic respiratory disease risk (13); long-term exposure to PM₁–₂.₅ was also positively associated with asthma incidence in middle-aged and older adults (14). Additionally, global COPD deaths attributable to ozone (O₃) among the elderly increased from 1,870 to 4,200 between 1990 and 2021 (15). Climate change, as another critical environmental risk, further threatens CLD patients by intensifying extreme temperature events (16). Despite global warming, cold spells remain frequent (17), and temperature-related morbidity and mortality are rising worldwide (18). Cold exposure consistently raises COPD mortality and hospitalization risks (19–21). This issue is particularly pronounced in China: about 14.33% of non-accidental deaths were attributable to non-optimal temperatures, with COPD accounting for 12.57% of this share (22). Consistently, an increased mortality risk for COPD related to cold spells was also observed in Hong Kong, China (23). Together, these findings highlight the importance of targeting environmental risks in CLD prevention and control. However, existing research has primarily focused on the impact of extreme temperatures on mortality from specific CLD such as COPD, with limited exploration of the spatial association patterns and multifactorial synergies affecting the overall incidence risk of CLD among China’s middle-aged and elderly population. Targeted studies are thus urgently needed to provide empirical evidence.

This study aims to systematically examine the association between cold spells and the incidence of CLD in Chinese adults aged 45 years and older, using a nationally representative sample. Leveraging five consecutive waves (2011–2020) of the CHARLS, we applied a time-to-event analytical approach. After adjusting for demographic characteristics, behavioral factors, clinical risk factors, and air pollution, we systematically assessed the effects of nine distinct cold spell indicators—defined by temperature thresholds and duration—on CLD incidence. Furthermore, we employed a GGPR model with a composite Matern kernel, combined with the GeoShapley method and permutation importance, to conduct a spatially explainable analysis. This approach not only explores the association between cold spell exposure and disease risk but also constructs a multidimensional feature-space machine learning model to predict CLD onset, quantify the contribution of characteristic variables, and identify spatial association patterns.

## 2 Methods

### 2.1 Study design and population

This study utilized data from the CHARLS (24). CHARLS employs a multistage probability sampling method to randomly select nationally representative samples of Chinese adults aged 45 years and older, aiming to provide high-quality microdata for research on aging. The baseline survey was conducted in 2011–2012, covering 17,708 individuals from 10,257 households across 450 communities in 150 counties. Follow-ups were conducted every 2 years using standardized questionnaires via face-to-face interviews. The survey collects information on demographics, family structure, economic status, health, physical measurements, healthcare utilization, insurance, employment, retirement, pensions, income, consumption, assets, and community characteristics. CHARLS has obtained ethical approval from the Institutional Review Board of Peking University (IRB00001052-11015) (24). All participants provided written informed consent. As this study involved secondary analysis of an established de-identified dataset, additional ethical approval and consent were not required.

**Figure 1:**
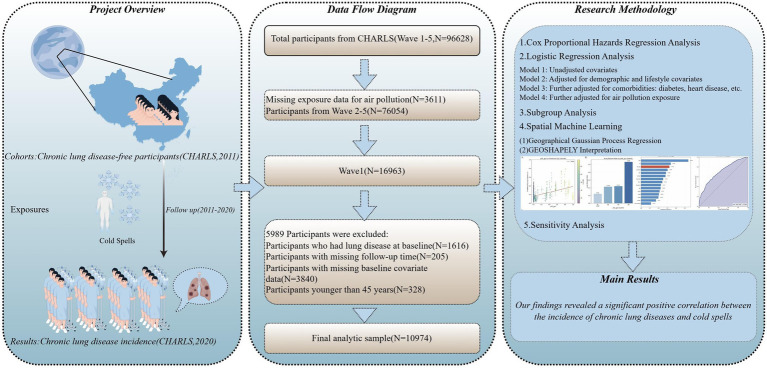
Flowchart of this study. Adapted with permission from “By Figdraw” platform.

This analysis drew on five CHARLS waves (2011, 2013, 2015, 2018, and 2020). The 2011–2020 window was selected because 2011 is the national baseline survey with the broadest coverage and highest response rate, while 2020 represents the most recent follow-up wave available at study initiation, thereby providing up to 9 years of follow-up for sufficient incident event accrual and aligning with the temporal coverage of available meteorological and air pollution data. Participant selection is detailed in Figure 1. First, individuals whose records could not be linked to air pollution metrics were excluded from the overall dataset (*n* = 3,611). From the remaining pool, baseline (2011) participants were identified (*n* = 16,963) and underwent further screening. Subsequent exclusions applied to this baseline cohort comprised individuals with pre-existing lung disease at baseline (*n* = 1,616), those aged below 45 years (*n* = 328), participants with missing key covariate data (*n* = 3,840), and individuals lost to follow-up (*n* = 205). The final analytical sample consisted of 10,974 participants.

### 2.2 Definitions of chronic lung diseases

We defined CLD as the primary outcome, ascertained through a specific CHARLS questionnaire item. Participants were asked: “Have you been diagnosed with the following chronic diseases by a doctor?” One listed item (Item 5 on Showcard 7) was “Chronic lung diseases, such as chronic bronchitis, emphysema, or other chronic lung diseases (excluding tumors or cancers) (25).” Affirmative responses were defined as indicative of the disease. Responses were operationalized as a binary variable (1 for presence, 0 for absence).

### 2.3 Definitions of extreme temperature events

Following an established approach (26), we defined 9 extreme temperature events by combining temperature thresholds and duration (Supplementary Table S1). Thresholds were set at the 7.5th, 5th, and 2.5th percentiles for cold spells. Event duration was defined as lasting ≥2, ≥3, or ≥4 consecutive days. Thus, a cold spell was defined analogously at or below the threshold. Data were obtained from the China Meteorological Administration Land Individuals were geographically matched to the 2011 extreme temperature data (based on residential city) to establish a fixed baseline exposure.

### 2.4 Definition of covariates

A comprehensive set of covariates was selected to control for potential confounding, including sociodemographic characteristics, lifestyle behaviors, clinical risk factors, and air pollutants (14, 25, 27). Sociodemographic variables comprised age, sex, residence (urban/rural), education (Below primary, Primary schools, Middle school, High school and above), and marital status (married, others). Lifestyle-related variables included smoking and drinking status (yes/no). Clinical risk factors included body mass index (BMI) and the presence of diabetes, cancer, heart disease, stroke, and disability. These data were obtained from CHARLS baseline questionnaires and physical measurements. Air pollutants (NO₂, O₃, PM₁, PM₂.₅, PM₁₀) were derived from the China High Air Pollutants (CHAP) dataset^1^ (14, 28, 29). All covariates, including air pollutants, were ascertained at baseline (2011) and treated as fixed values throughout the follow-up period.

### 2.5 Statistical analysis

Descriptive analyses were performed for baseline characteristics. Categorical variables were presented as frequencies and proportions, and compared using the chi-square test. Continuous variables were summarized as mean and standard deviation (SD) if normally distributed, or as median and interquartile range (IQR) otherwise. The Mann–Whitney U test was used for group comparisons.

To systematically assess the association between baseline cold spells exposure and the incidence of CLD among middle-aged and older adults, we employed two complementary statistical approaches. The primary analysis used Cox proportional hazards models for time-to-event analysis. Hazard ratios (HRs) with 95% confidence intervals (CIs) were calculated to assess risk, and the proportional hazards assumption was examined using Schoenfeld residuals. Additionally, logistic regression analysis was performed as a supplementary method, modeling the binary outcome of CLD presence by the end of follow-up (2020) to generate odds ratios (ORs) with 95% CIs. Both analytical approaches utilized the same four nested models with sequential covariate adjustment to further evaluate the robustness of the associations and account for potential confounding. Model 1 was a crude model. Model 2 was adjusted for age, gender, marital status, educational level, residence, smoking and drinking status, and BMI. Subsequently, Model 3 was further adjusted for comorbidities (diabetes, cancer, heart disease, stroke and disability). Finally, Model 4 represented the fully adjusted model, which additionally incorporated air pollutants. Then, subgroup analyses were conducted by age, gender, marital status, educational level, residence, smoking and drinking status, BMI, and the presence of five chronic comorbidities to assess heterogeneity and test for subgroup differences using interaction terms. Given that subgroup analyses involved multiple cold spell definitions and several subgroup variables, the Benjamini-Hochberg procedure was applied to control the false discovery rate (FDR) across all interaction tests, yielding adjusted *q*-values. Statistical significance for interaction terms was defined as *q* < 0.05.

Furthermore, we employed GGPR to investigate the spatial association between cold spells and CLD. This method integrates Gaussian process machine learning with geospatial analysis to effectively capture spatial autocorrelation, while the GeoShapley function provides interpretable feature importance metrics. To further evaluate the incremental contribution of geographic coordinates and to assess potential overfitting in the spatial prediction models, we constructed three nested model specifications: M0 (geographic coordinates only; i.e., longitude and latitude), M1 (cold spell exposure plus all covariates, excluding coordinates), and M2 (full model: exposure + covariates + coordinates). Model performance was evaluated using city-grouped 5-fold cross-validation (GroupKFold), whereby all samples from the same city were assigned to the same fold to prevent spatial information leakage and to provide a more realistic assessment of generalizability to unseen locations. Area under the receiver operating characteristic curve (AUC) and Brier scores were computed for each model. In addition, permutation importance was used to rank feature contributions in M1 and M2, enabling assessment of changes in the relative importance of cold spell exposure after accounting for spatial coordinates. To evaluate the consistency of city-level cold spell exposure over the 10-year follow-up period, we calculated the intraclass correlation coefficient (ICC) for annual cold spell days across all included cities from 2011 to 2020. A two-way mixed-effects model for absolute agreement was used to compute both single-measures ICC (reflecting the reliability of a single year’s measurement) and average-measures ICC (reflecting the reliability of the 10-year average). F-tests were performed to assess the statistical significance of the ICC values, with a *p*-value <0.05 considered significant. This analysis aimed to quantify the extent to which baseline (2011) cold spell exposure serves as a reliable proxy for long-term exposure ranking. All statistical analyses were performed using R software (version 4.4.1) and the GGPR Analysis Tool v2.0. Two-sided tests were applied, with a *p*-value <0.05 considered statistically significant.

### 2.6 Sensitivity analysis

Sensitivity analyses were carried out to evaluate the robustness of the results:

(1) Standardization of continuous variables: All continuous variables, including both covariates and exposure measures, were standardized using Z-scores to unify their scales and mitigate the influence of extreme values before being re-entered into the Cox models.
(2) Analyses at alternative follow-up times: In order to assess the impact of follow-up duration, the main models were repeated with follow-up censored at Wave 3 (2015) and Wave 4 (2018).
(3) Exclusion of baseline asthma cases: To limit potential reverse causation, analyses excluded participants who reported asthma at baseline and the models were re-fitted accordingly.
(4) Exclusion of the cancer history subgroup: Given the relatively limited sample size of participants with a self-reported history of cancer, the fully adjusted Cox models were re-fitted after excluding this subgroup to verify that the main effect estimates were not unduly influenced by this specific subpopulation.

All sensitivity analyses applied the same sequential covariate adjustment strategy using four nested models as in the primary analysis.

## 3 Results

### 3.1 Population characteristics

The baseline characteristics were summarized in Table 1. A total of 10,974 participants free of the target CLD at baseline were included in this study. By the end of follow-up, 1,587 individuals (14.5%) had developed new-onset CLD. Compared with the non-disease group, the CLD group had significantly higher proportions of urban residence (42.2% vs. 36.1%), alcohol drinking history (41.5% vs. 37.9%), smoking history (42.7% vs. 37.8%), and pre-existing heart disease (13.9% vs. 10.2%) (all *p* < 0.05). Regarding environmental exposure, the incident CLD group showed significantly lower median baseline concentrations for all five air pollutants (NO₂, O₃, PM₁, PM₂.₅, PM₁₀) (all *p* < 0.001). In addition, no significant differences were observed between the two groups in age, gender, education level, marital status, BMI, or prevalence of diabetes, cancer, stroke, and disability (all *p* > 0.05).

**Table 1:** Characteristics of the participants at baseline.

Characteristic	Overall, *N* = 10,974	Lung disease		*p*-value^1^
		No, *N* = 9,387	Yes, *N* = 1,587	
Age, years, Median (IQR)	58.00 (51.00, 65.00)	58.00 (51.00, 65.00)	58.00 (52.00, 65.00)	0.174
Education, *n* (%)				0.197
Below primary	5,139.0 (46.8%)	4,398.0 (46.9%)	741.0 (46.7%)	
Primary schools	2,355.0 (21.5%)	2,040.0 (21.7%)	315.0 (19.8%)	
Middle school	2,267.0 (20.7%)	1,929.0 (20.5%)	338.0 (21.3%)	
High school and above	1,213.0 (11.1%)	1,020.0 (10.9%)	193.0 (12.2%)	
Gender, *n* (%)				0.084
Male	5,098.0 (46.5%)	4,329.0 (46.1%)	769.0 (48.5%)	
Female	5,876.0 (53.5%)	5,058.0 (53.9%)	818.0 (51.5%)	
Marry, *n* (%)				0.955
Yes	9,582.0 (87.3%)	8,197.0 (87.3%)	1,385.0 (87.3%)	
Others	1,392.0 (12.7%)	1,190.0 (12.7%)	202.0 (12.7%)	
Residence, *n* (%)				<0.001
Rural	6,913.0 (63.0%)	5,996.0 (63.9%)	917.0 (57.8%)	
Urban	4,061.0 (37.0%)	3,391.0 (36.1%)	670.0 (42.2%)	
Alcohol drinking status, *n* (%)				0.007
Yes	4,220.0 (38.5%)	3,561.0 (37.9%)	659.0 (41.5%)	
No	6,754.0 (61.5%)	5,826.0 (62.1%)	928.0 (58.5%)	
Smoking status, *n* (%)				<0.001
Yes	4,229.0 (38.5%)	3,551.0 (37.8%)	678.0 (42.7%)	
No	6,745.0 (61.5%)	5,836.0 (62.2%)	909.0 (57.3%)	
BMI, kg/m^2: Median (IQR)	23.18 (20.89, 25.76)	23.17 (20.87, 25.78)	23.28 (20.97, 25.74)	0.674
Diabetes, *n* (%)				0.658
Yes	656.0 (6.0%)	565.0 (6.0%)	91.0 (5.7%)	
No	10,318.0 (94.0%)	8,822.0 (94.0%)	1,496.0 (94.3%)	
Cancer, *n* (%)				0.194
Yes	94.0 (0.9%)	76.0 (0.8%)	18.0 (1.1%)	
No	10,880.0 (99.1%)	9,311.0 (99.2%)	1,569.0 (98.9%)	
Heart Disease, *n* (%)				<0.001
Yes	1,179.0 (10.7%)	958.0 (10.2%)	221.0 (13.9%)	
No	9,795.0 (89.3%)	8,429.0 (89.8%)	1,366.0 (86.1%)	
Stroke, *n* (%)				0.798
Yes	253.0 (2.3%)	215.0 (2.3%)	38.0 (2.4%)	
No	10,721.0 (97.7%)	9,172.0 (97.7%)	1,549.0 (97.6%)	
Disability, *n* (%)				0.136
Yes	1,883.0 (17.2%)	1,590.0 (16.9%)	293.0 (18.5%)	
No	9,091.0 (82.8%)	7,797.0 (83.1%)	1,294.0 (81.5%)	
NO2, Median (IQR)	28.44 (22.09, 37.26)	28.66 (21.63, 38.11)	25.75 (22.21, 31.04)	<0.001
O3, Median (IQR)	84.47 (81.34, 88.91)	84.82 (81.54, 89.25)	83.29 (80.66, 86.73)	<0.001
PM10, Median (IQR)	97.41 (66.84,119.27)	98.05 (65.76,122.29)	86.93 (74.92,112.88)	<0.001
PM1, Median (IQR)	31.34 (25.24, 38.80)	31.34 (24.87, 39.38)	31.22 (25.24, 36.79)	<0.001
PM2.5, Median (IQR)	55.25 (42.33, 71.19)	55.98 (41.78, 71.34)	53.13 (45.25, 69.73)	<0.001

^1^Wilcoxon rank sum test; Pearson’s Chi-squared test. IQR, interquartile range; BMI, body mass index; PM1, Particulate matter 1; PM2.5, Particulate matter 2.5; PM10, Particulate matter 10; NO2, Nitrogen dioxide.

### 3.2 Temporal stability of cold spell exposure

The ICC analysis demonstrated excellent year-to-year consistency of cold spell exposure at the city level across the 10-year study period. Single-measures ICC values ranged from 0.847 to 0.876 across individual years, while average-measures ICC values ranged from 0.980 to 0.985 (Supplementary Table S2). All F-test *p*-values were <0.001, indicating that the vast majority of the variance in cold spell exposure was attributable to between-city differences rather than year-to-year fluctuations. These findings support the use of baseline cold spell exposure as a reliable proxy for long-term exposure ranking in the subsequent longitudinal analyses.

### 3.3 The association between cold spells and incident CLD

This study employed Cox proportional hazards models and logistic regression models to systematically evaluate the association between cold spells and incident CLD in middle-aged and older adults. Cox regression analysis revealed a significant positive association between cold spells and disease risk across all nine definitions (Table 2). In the unadjusted model (Model 1), the HRs ranged from 1.06 to 1.09, indicating a 6–9% increase in risk (all *p* < 0.001). Adjustment for sociodemographic characteristics, lifestyle factors, and BMI (Model 2) yielded stable HRs (1.06–1.09). Further adjustment for comorbidities (Model 3) did not substantially alter the estimates. In the fully adjusted model (Model 4, which additionally included air pollutants), the association was strengthened, with HRs increasing to 1.10–1.13, representing a 10–13% higher risk (all *p* < 0.001). Notably, within the fully adjusted model, risk estimates exhibited a modest, non-linear increase with longer spell duration under the same temperature threshold (e.g., HR increased from 1.12 for ≥2 days to 1.13 for ≥4 days at the 5th percentile). Conversely, varying the temperature percentile threshold (e.g., from the 7.5th to the 2.5th percentile) while holding duration constant resulted in only minimal fluctuations in risk estimates. Logistic regression results were consistent (Supplementary Table S3). The unadjusted ORs were 1.07–1.10 (equivalent to a 7–10% increase in odds; all *p* < 0.001). Subsequent adjustments in Model 2 (ORs: 1.06–1.09) and Model 3 showed similar estimates. In the fully adjusted Model 4, ORs further increased to 1.11–1.13 (an 11–13% increase in odds; all *p* < 0.001). These consistent findings indicate that cold spell is an independent risk factor for CLD, associated with an approximately 10–13% increase in risk in the fully adjusted Cox model.

**Table 2:** Associations of cold spells with CLD.

Characteristic	Model 1		Model 2		Model 3		Model 4	
	HR (95%CI)	*P*	HR (95%CI)	*P*	HR (95%CI)	*P*	HR (95%CI)	*P*
p075_ge2	1.06 (1.05,1.08)	<0.001	1.06 (1.05,1.08)	<0.001	1.06 (1.05,1.07)	<0.001	1.11 (1.09,1.13)	<0.001
p075_ge3	1.06 (1.05, 1.07)	<0.001	1.06 (1.05, 1.07)	<0.001	1.06 (1.04, 1.07)	<0.001	1.10 (1.09, 1.12)	<0.001
p075_ge4	1.06 (1.05, 1.08)	<0.001	1.06 (1.05, 1.07)	<0.001	1.06 (1.05, 1.07)	<0.001	1.10 (1.09, 1.12)	<0.001
p05_ge2	1.08 (1.06, 1.09)	<0.001	1.08 (1.06, 1.09)	<0.001	1.07 (1.06, 1.09)	<0.001	1.12 (1.10, 1.14)	<0.001
p05_ge3	1.08 (1.07, 1.09)	<0.001	1.08 (1.06, 1.09)	<0.001	1.07 (1.06, 1.09)	<0.001	1.12 (1.10, 1.14)	<0.001
p05_ge4	1.08 (1.07, 1.10)	<0.001	1.08 (1.07, 1.10)	<0.001	1.08 (1.06, 1.09)	<0.001	1.13 (1.11, 1.15)	<0.001
p025_ge2	1.08 (1.06, 1.10)	<0.001	1.08 (1.06, 1.10)	<0.001	1.07 (1.06, 1.09)	<0.001	1.11 (1.09, 1.14)	<0.001
p025_ge3	1.09 (1.07, 1.11)	<0.001	1.09 (1.07, 1.11)	<0.001	1.08 (1.06, 1.10)	<0.001	1.13 (1.10, 1.15)	<0.001
p025_ge4	1.09 (1.07, 1.11)	<0.001	1.08 (1.06, 1.11)	<0.001	1.08 (1.06, 1.10)	<0.001	1.12 (1.09, 1.15)	<0.001

Model 1: crude model. Model 2: adjusting age, gender, marital status, educational level, residence, smoking and drinking status, and BMI. Model 3: Model 2 + additional adjusting comorbidities (diabetes, cancer, heart disease, stroke and disability). Model 4: Model 3 + additional adjusting air pollutants. HR, hazard ratio; CI, confidence interval.

### 3.4 Subgroup analysis of cold spells and the risk of CLD

**Figure 2:**
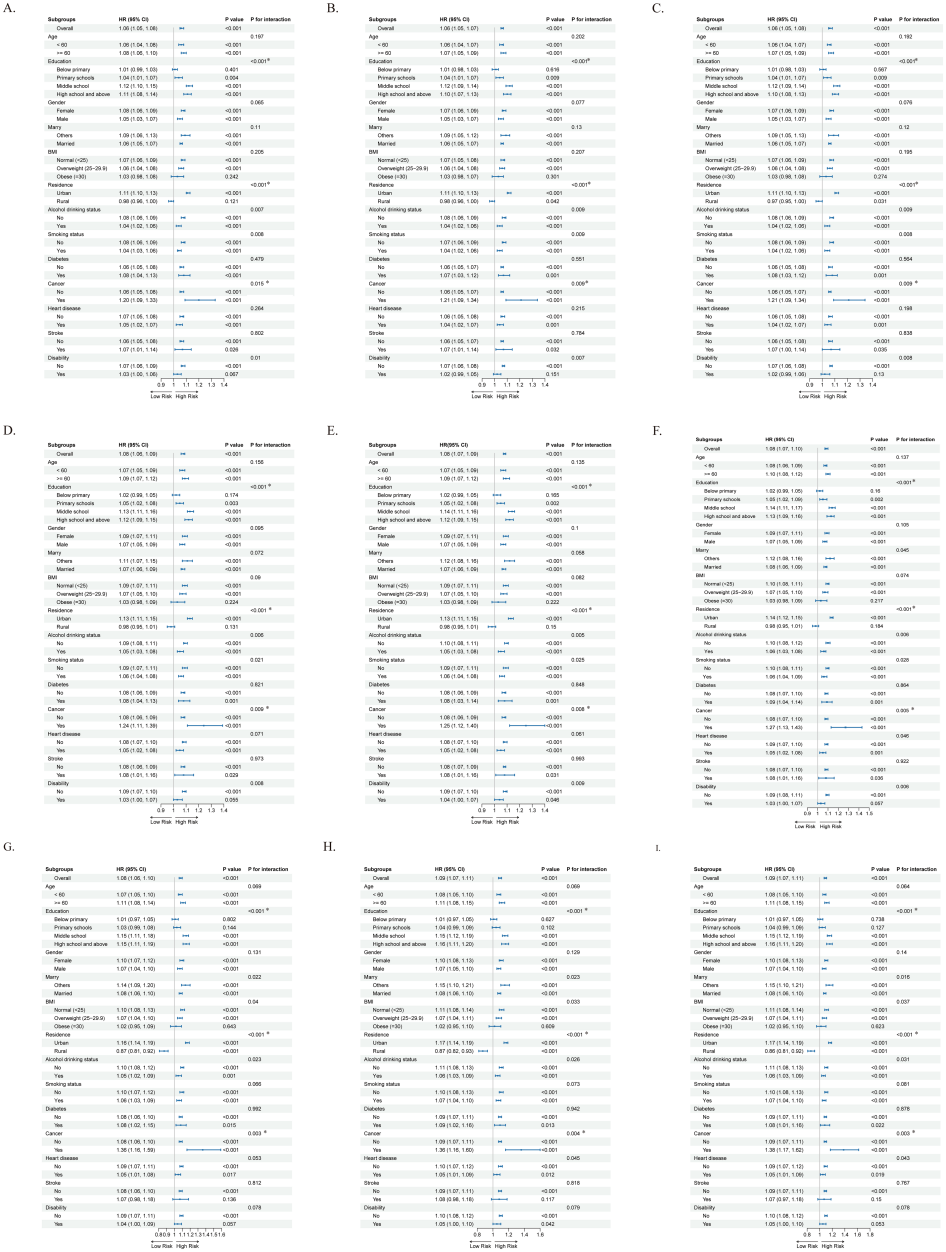
Subgroup analysis of cold spells and the risk of CLD. **(A)** p075_ge2; **(B)** p075_ge3; **(C)** p075_ge4; **(D)** p05_ge2; **(E)** p05_ge3; **(F)** p05_ge4; **(G)** p025_ge2; **(H)** p025_ge3; **(I)** p025_ge4. Hazard ratios (HRs) and 95% confidence intervals are displayed. Asterisks (*) denote subgroup interactions that remained statistically significant after Benjamini-Hochberg false discovery rate (FDR) correction (*q* < 0.05).

Subgroup analyses revealed notable population heterogeneity in the association between cold spells and CLD risk, with effect sizes significantly modified by education level, residence, and cancer history (nominal interaction *p* < 0.05, Figure 2). These interactions remained statistically significant after Benjamini-Hochberg FDR correction (Supplementary Table S4, adjusted q < 0.05; denoted by asterisks in Figure 2). Specifically, the risk increased with higher education levels, reaching an HR of 1.16 among individuals with high school or above education, whereas no significant association was observed in those with primary school or lower education. The elevated risk was mainly concentrated in urban residents (HRs = 1.11–1.17), with no significant increase observed in rural residents. Patients with a history of cancer exhibited further elevated risk, with HRs ranging from 1.20 to 1.38. Notably, although the interaction for cancer history remained significant after FDR correction (q-values ranging from 0.0176 to 0.0418), this subgroup comprised only 94 participants with 18 incident CLD events; therefore, this finding should be regarded as exploratory and warrants validation in larger studies. Collectively, these findings suggest that urban residents, those with higher education levels, and individuals with a cancer history may represent potentially vulnerable subgroups to cold spells. The observed heterogeneity further indicates that the health impacts of cold spells may be closely related to socioeconomic background, behavioral adaptability, and environmental characteristics.

### 3.5 Sensitivity analysis

To assess the robustness of the primary findings, a series of sensitivity analyses were conducted (see Supplementary Tables S5–S9). After standardizing continuous variables using z-score transformation, the effect pattern of cold spells on CLD risk—namely, an increased risk of CLD—and its statistical significance remained unchanged, indicating robust results. Next, by shortening the follow-up period to 2015 or 2018, the harmful association of cold spells remained significant, suggesting that the findings were replicable across different observation periods. To control for potential reverse causation, individuals with diagnosed asthma at baseline were excluded; the results continued to show a significant positive association between cold spells and incident CLD. Finally, to verify that the relatively small cancer history subgroup (*n* = 94) did not unduly influence the main effect estimates, the fully adjusted Cox models were re-fitted after excluding these participants. The association between cold spell exposure and incident CLD remained statistically significant across all nine definitions, with negligible changes in hazard ratios, further supporting the reliability of the main conclusions.

### 3.6 Spatial machine learning

**Figure 3:**
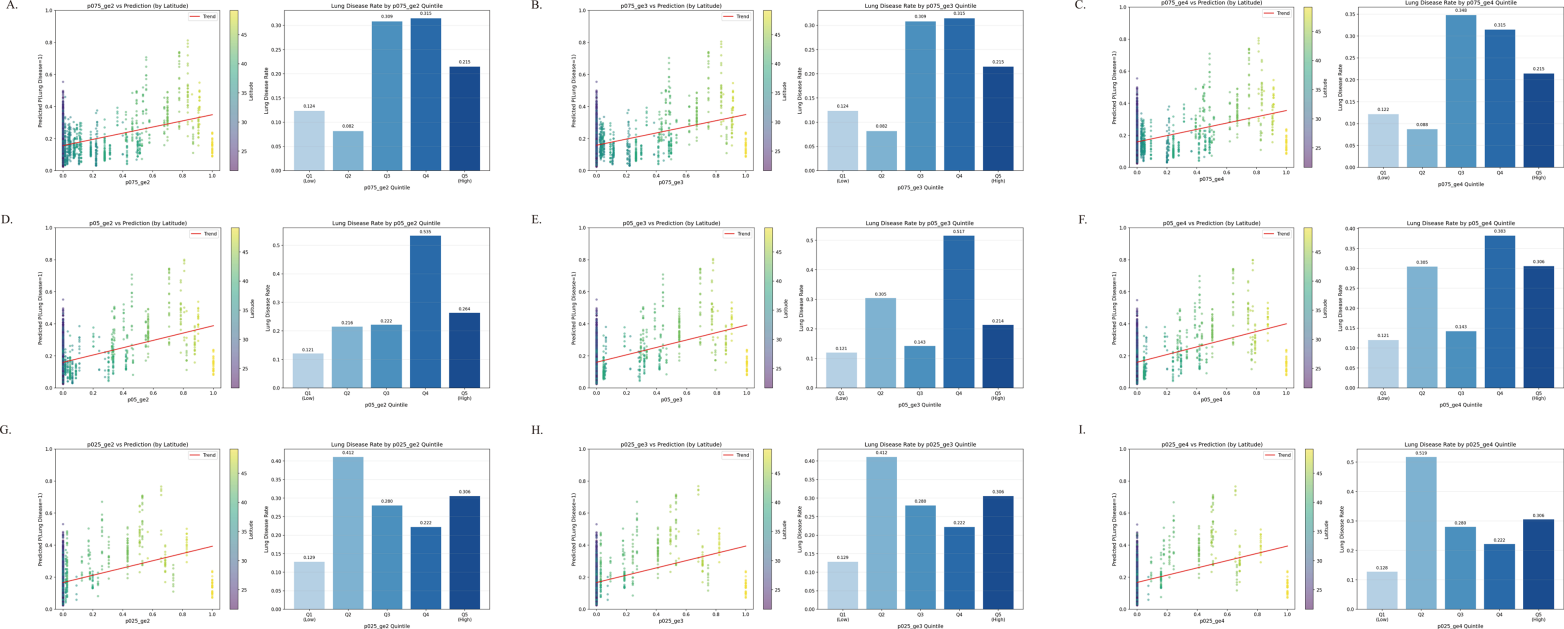
Exposure–response curve analysis for cold spells based on the spatial machine learning model. **(A)** p075_ge2; **(B)** p075_ge3; **(C)** p075_ge4; **(D)** p05_ge2; **(E)** p05_ge3; **(F)** p05_ge4; **(G)** p025_ge2; **(H)** p025_ge3; **(I)** p025_ge4.

The spatial machine learning model incorporated cold spell exposure metrics, geographic coordinates (longitude and latitude), demographic characteristics, behavioral factors, BMI, history of comorbidities, and air pollutant concentrations as covariates to capture the non-stationarity of spatial structure. According to the exposure–response curve analysis from the spatial machine learning approach, the effect of cold spells on CLD risk exhibited a nonlinear relationship (Figure 3). Taking the cold spell indicator p05-ge2 as an example, a positive association was observed between exposure level and disease risk, with the highest CLD incidence (0.535) occurring at the Q4 quantile. Notably, the incidence then fell to 0.264 at the Q5 quantile. This non-monotonic trend further indicates a complex dose–response relationship between cold spells and CLD (Figure 3D). The GGPR-based spatial prediction model demonstrated stable performance across all cold spell exposure definitions (Supplementary Figure S1). The AUC values for all models in the validation set ranged from 0.7077 to 0.7129, indicating moderate and consistent discriminative ability (Supplementary Table S10). Model stability was reflected in the following aspects: the mean AUC difference between the training and validation sets was only 0.017 (0.729 vs. 0.712), well below the threshold typically suggestive of overfitting; the standard deviation of validation AUC across different exposure indicators was only 0.002, indicating minimal fluctuation in predictive performance; furthermore, varying the continuous exposure duration threshold (2, 3, or 4 days) had negligible impact on AUC (difference < 0.003).

To further evaluate generalizability and address potential spatial information leakage, we performed nested model comparisons with city-grouped cross-validation (GroupKFold) (Supplementary Table S11). The coordinate-only model (M0) exhibited near-random performance (GroupKFold AUC ≈ 0.55 across definitions), confirming that geographic coordinates alone provide negligible predictive information. The exposure-plus-covariates model (M1) achieved GroupKFold AUCs ranging from 0.5546 to 0.5958, demonstrating transportable predictive value. Addition of coordinates in the full model (M2) yielded only marginal improvements (ΔAUC≤0.025), with Brier scores virtually unchanged. Permutation importance analysis showed that cold spell exposure ranked among the top predictors in M1 (e.g., 3rd for p075_ge2) and remained within the top ten in M2. Independent test set validation yielded consistent findings (Supplementary Table S12). Notably, the lower GroupKFold AUC of M2 (~0.60) relative to the randomly split validation AUC (~0.71) is expected: random splitting may overestimate generalizability due to spatial autocorrelation, whereas GroupKFold provides a stricter, more realistic assessment by holding out entire cities. Critically, M2 still outperformed M0 under this rigorous evaluation, confirming that cold spell exposure retains independent, transportable predictive value beyond regional background effects.

Feature attribution based on the GeoShapley method indicated that although cold spell exposure contributed significantly, its importance was lower than that of key covariates such as air pollutants (NO₂, O₃, PM₁) and geographic coordinates, suggesting that the effect of extreme temperature operates within a multifactorial context (Supplementary Figure S2). Spatial prediction results showed that the high-risk areas identified by the model (longitude > 100, latitude > 30) closely aligned with the actual distribution of disease incidence (Supplementary Figure S3). The core high-risk zone was concentrated within longitude ≥ 110 and latitude 35–50. Geographically, high-risk cities were predominantly located in northern China, especially within the three northeastern provinces and the five northern provinces, exhibiting a north-to-south declining risk gradient that corresponded closely with the color gradient in the model prediction map. Although different cold spell indicators yielded similar spatial prediction patterns, this is expected because the dominant spatial structure was primarily driven by geographic location and stable covariates. Different exposure definitions mainly affected individual-level predicted probabilities rather than reshaping the overall spatial risk pattern.

## 4 Discussion

This study provides comprehensive evidence linking cold spells to an elevated risk of CLD incidence among adults aged ≥45 years in China. Multiple analytical approaches consistently identified this association, which remained significant after extensive adjustment for demographic, behavioral, clinical, and environmental confounders. Urban residents, those with higher education levels, and cancer survivors were particularly vulnerable. GGPR analysis further revealed a nonlinear dose–response relationship, and the spatial prediction model performed stably across varying cold spell definitions. Sensitivity analyses supported the robustness of these findings.

The findings demonstrate that all cold spell metrics were positively associated with CLD incidence. This association aligns with prior evidence on the adverse effects of cold on CLD. Extreme cold may synergize with respiratory viral infections to worsen disease outcomes by exacerbating airway inflammation (30, 31). Mechanistically, cold exposure can induce acute bronchoconstriction and airflow limitation, impair mucociliary clearance, and compromise airway epithelial barrier integrity, thereby contributing to the development and progression of CLD (32–36). Environmental co-exposures, such as low humidity and elevated air pollutant concentrations during cold spells, may further amplify airway inflammation and oxidative stress (37–39). However, a previous CHARLS-based study reported no significant association between extreme temperatures and CLD incidence among older adults (25), a discrepancy that may reflect differences in exposure definitions, outcome ascertainment, or the possibility that cold primarily triggers acute exacerbations rather than disease onset.

Our stratified analysis suggests that the association between cold spells and CLD risk is significantly modified by education level, residence, and cancer history, with higher risks observed in urban residents, individuals with higher education, and cancer survivors. This finding aligns with existing research on population disparities in climate vulnerability. First, the elevated risk among urban residents may be attributed to factors such as the urban heat island effect, which amplifies temperature fluctuations during cold spells, and to synergistic interactions between cold and urban air pollution, potentially worsening respiratory inflammation and oxidative stress (20, 40, 41). Additionally, urban lifestyles involving frequent indoor-outdoor transitions may also increase cold air exposure. Second, the higher risk in more educated individuals may reflect a greater likelihood of diagnosis (diagnostic bias), as this group often has greater health awareness, better healthcare access, and higher exposure to urban air pollution, all of which can lead to increased detection of lung diseases (42, 43). Residual confounding from unmeasured factors, such as specific indoor occupational exposures (e.g., to substances from office ventilation systems), may also contribute. Finally, cancer survivors, particularly those with prior thoracic radiotherapy or pneumotoxic chemotherapy, often have reduced lung reserve and heightened airway responsiveness, making them more vulnerable to environmental stressors such as cold and thereby increasing long-term respiratory morbidity (44, 45). These findings highlight the complexity of climate-health interactions and underscore the need for tailored public health strategies that address population-specific vulnerabilities. Recommended measures include mitigating the urban heat island effect, controlling the compounded risk of air pollution during cold spells, and improving the urban microclimate and air quality. Furthermore, establishing specialized lung health management programs for cancer survivors, to provide personalized protection guidance and medical support based on their lung function, is essential. Attention should also be paid to potential diagnostic bias and to occupational exposures in highly educated groups. These measures are crucial for enhancing population resilience against extreme temperatures.

This study employed the GGPR model combined with the GeoShapley feature analysis method to systematically examine the spatial association between cold spell exposure and CLD incidence. Results showed a significant positive association, consistent with Global Burden of Disease findings on respiratory risks from low temperatures. The GGPR model demonstrated stable performance, effectively addressing the limitation of traditional time-series models in quantifying large-scale spatial heterogeneity and outperforming traditional models like geographically weighted regression in multi-covariate integration. GeoShapley analysis revealed that contributions from air pollutants (NO_2_, O_3_, PM_1_) and geographic coordinates exceeded that of cold exposure itself. This confirmed that the impact of cold spells on lung disease must be understood within the complex system of “environmental pollutants – spatial heterogeneity – individual exposure,” echoing multinational research on the close link between particulate matter exposure and respiratory diseases (46). Spatial prediction showed a “high in the north, low in the south” gradient in lung disease risk, with high-risk areas concentrated in Northeast and North China. This pattern corresponds to the geographic distribution of extreme cold-related health burdens reported by the Intergovernmental Panel on Climate Change (IPCC) and matches key areas identified in Zhang et al.’s spatial study on cold-related respiratory disease burden in China (47, 48). On a global scale, Gasparrini et al.’s Lancet study covering over 750 cities in 13 countries also identified mid-to-high latitude regions in the Northern Hemisphere as core hotspots for cold-related health risks (49). Based on these findings and relevant practices, we propose the following public health recommendations: (1) Establish a “cold spell-air pollution” compound early warning system in high-risk areas, promoting tiered regional responses with reference to frameworks like the US Environmental Protection Agency’s risk assessment guidelines (50); (2) Strengthen pollution control and urban adaptation planning in key northern regions, and enhance CLD screening and management for vulnerable groups like the elderly; (3) Strengthen inter-agency coordination and risk-specific public health education to improve community resilience.

This study has several strengths. First, it focuses on adults aged ≥45 years — a susceptible population — and examines the association between cold spells and overall CLD incidence, thereby balancing general disease characteristics with specific risk assessment and enhancing public health relevance. Second, the analysis comprehensively adjusted for a wide range of potential confounders, including demographic, behavioral, clinical, and air pollution covariates. Methodologically, the integration of the GGPR model with GeoShapley analysis represents a key advance. This approach not only captured large-scale spatial heterogeneity from an incidence perspective but also quantified the relative contributions of different factors, thereby improving both spatial risk prediction and mechanistic interpretability.

This study has several limitations. First, due to the lack of detailed data on indoor air pollution (e.g., household solid fuel use), occupational factors, and individual-level cold spell exposure, residual confounding may exist. Second, cold spell exposure was assigned based on residential city at baseline (2011) and assumed to remain constant throughout the nine-year follow-up. Although supplementary ICC analysis indicated high year-to-year stability of city-level cold spell exposure, thereby supporting the use of baseline exposure as a reasonable proxy for long-term ranking, this approach does not capture potential within-period changes in exposure due to residential mobility or interannual climatic variability. Third, spatially, the analysis was conducted at a national scale and could not capture within-city heterogeneity, limiting insights into local clustering. Fourth, the absence of a standardized cold-spell definition means results may vary across indicators, and we did not assess potential differential responses across CLD subtypes. Fifth, because the CLD outcome was based on self-reported physician diagnosis, we cannot fully exclude the possibility that some cases represent newly detected—rather than truly new-onset—disease. If cold spells prompt previously undiagnosed individuals to seek medical attention, the resulting non-differential misclassification would likely bias effect estimates conservatively toward the null.

## 5 Conclusion

In this nationwide cohort study of Chinese adults aged ≥45 years, higher baseline cold spell exposure was associated with a modestly increased risk of incident CLD. Northern China was consistently identified as a high-risk region across all cold spell definitions. While GeoShapley analysis indicated that air pollutants and geographic coordinates contributed more substantially to spatial prediction, the independent longitudinal association of cold spells persisted after covariate adjustment, underscoring their potential relevance as an environmental trigger. These findings may inform region-specific public health strategies for protecting vulnerable aging populations under climate variability.

## Data Availability

The data underlying this study are publicly available from the CHARLS official website (http://charls.pku.edu.cn/).

## Footnotes

1
https://weijing-rs.github.io/product.html

## Glossary

CLD: Chronic lung disease
CHARLS: China Health and Retirement Longitudinal Study
GGPR: Geographical Gaussian Process Regression
COPD: Chronic obstructive pulmonary disease
DALYs: Disability-adjusted life years
BMI: Body mass index
PM1: Particulate matter 1
PM2.5: Particulate matter 2.5
PM10: Particulate matter 10
NO2: Nitrogen dioxide
CHAP: China High Air Pollutants
HR: Hazard ratio
OR: Odds ratio
CI: Confidence interval
SD: Standard deviation
IQR: Interquartile range
IPCC: Intergovernmental Panel on Climate Change
FDR: False discovery rate
AUC: Area under the receiver operating characteristic curve
ICC: Intraclass correlation coefficient
