# Supplementary material for: Association between cold spells and chronic lung disease: a nationwide spatial machine learning analysis
Source: Front Med (Lausanne). 2026 May 13;13:1823044. doi: 10.3389/fmed.2026.1823044 (PMC13212537; doi:10.3389/fmed.2026.1823044)
Supplement: Supplementary file 1 [file Data_sheet_1.pdf]

Supplementary materials

Table S1 Definition of cold spell.

Table S2 Intraclass correlation coefficients (ICC) for annual cold spell days across Chinese cities, 2011–2020.

Table S3 Associations of cold spells with CLD by Logistic regression analysis.

Table S4 False discovery rate (FDR) adjusted q-values for subgroup interaction tests across nine cold spell definitions

Table S5 Sensitivity analysis of the effect of cold spells on CLD risk after z-score standardization of continuous variables.

Table S6 Sensitivity analysis of the effect of cold spells on CLD risk after shortening the follow-up period to Wave 3.

Table S7 Sensitivity analysis of the effect of cold spells on CLD risk after shortening the follow-up period to Wave 4.

Table S8 Sensitivity analysis of the effect of cold spells on CLD risk after excluding participants with diagnosed asthma at baseline.

Table S9 Sensitivity analysis of the effect of cold spells on CLD risk after excluding participants with diagnosed cancer at baseline

Table S10 ROC analysis results of the spatial machine learning model across different cold-spell indicators.

Table S11 Spatial cross-validation performance of nested spatial prediction models across nine cold spell definitions.

Table S12 Independent test set performance of M1 and M2 models across cold spell definitions.

Figure. S1 ROC analysis results of the spatial machine learning model across different cold-spell indicators.

Figure. S2 Feature importance attribution for cold spells using the GeoShapley method in spatial machine learning.

Figure. S3 Cold-Spell Indicators: Spatial maps of the machinel earning model results.

Table S1 Definition of cold spell.

Definitions	Threshold	Duration	Intensity
P075_ge2	7.5th	≥ 2	Low
P075_ge3	7.5th	≥ 3	Low
P075_ge4	7.5th	≥ 4	Low
P05_ge2	5th	≥ 2	Middle
P05_ge3	5th	≥ 3	Middle
P05_ge4	5th	≥ 4	Middle
P025_ge2	2.5th	≥ 2	High
P025_ge3	2.5th	≥ 3	High
P025_ge4	2.5th	≥ 4	High

Table S2 Intraclass correlation coefficients (ICC) for annual cold spell days across Chinese cities, 2011–2020.

Year	Measures	ICCb	95% Confidence Interval		F Test with True Value 0			
			Lower	Upper	Value	df1	df1	*p*-value
2011	Single Measures	.872a	0.854	0.889	62.326	369	2952	0
	Average Measures	.984c	0.981	0.986	62.326	369	2952	0
2012	Single Measures	.865a	0.847	0.883	58.75	369	2952	0
	Average Measures	.983c	0.98	0.985	58.75	369	2952	0
2013	Single Measures	.875a	0.858	0.892	64.161	369	2952	0
	Average Measures	.984c	0.982	0.987	64.161	369	2952	0
2014	Single Measures	.855a	0.836	0.874	54.251	369	2952	0
	Average Measures	.982c	0.979	0.984	54.251	369	2952	0
2015	Single Measures	.865a	0.846	0.883	58.655	369	2952	0
	Average Measures	.983c	0.98	0.985	58.655	369	2952	0
2016	Single Measures	.876a	0.859	0.893	64.778	369	2952	0
	Average Measures	.985c	0.982	0.987	64.778	369	2952	0
2017	Single Measures	.849a	0.829	0.869	51.656	369	2952	0
	Average Measures	.981c	0.978	0.983	51.656	369	2952	0
2018	Single Measures	.867a	0.849	0.884	59.693	369	2952	0
	Average Measures	.983c	0.981	0.986	59.693	369	2952	0
2019	Single Measures	.847a	0.827	0.867	50.997	369	2952	0
	Average Measures	.980c	0.977	0.983	50.997	369	2952	0
2020	Single Measures	.854a	0.834	0.873	53.757	369	2952	0
	Average Measures	.981c	0.978	0.984	53.757	369	2952	0

ICC, intraclass correlation coefficient; CI, confidence interval; df, degrees of freedom.

Single measures ICC reflects the reliability of a single year's measurement; average measures ICC reflects the reliability of the 10-year average. ICCs were calculated using a two-way mixed-effects model for absolute agreement. All p-values were derived from F-tests for the null hypothesis that ICC = 0.

Table S3 Associations of cold spells with CLD by Logistic regression analysis.

Variables	Model 1		Model 2		Model 3		Model 4	
	OR (95%CI)	*P*	OR (95%CI)	*P*	OR (95%CI)	*P*	OR (95%CI)	*P*
P075_ge2	1.07 (1.06 ~ 1.08)	**<.001**	1.07 (1.05 ~ 1.08)	**<.001**	1.07 (1.05 ~ 1.08)	**<.001**	1.12 (1.10 ~ 1.14)	**<.001**
P075_ge3	1.07 (1.05 ~ 1.08)	**<.001**	1.06 (1.05 ~ 1.08)	**<.001**	1.06 (1.05 ~ 1.08)	**<.001**	1.11 (1.09 ~ 1.13)	**<.001**
P075_ge4	1.07 (1.05 ~ 1.08)	**<.001**	1.06 (1.05 ~ 1.08)	**<.001**	1.06 (1.05 ~ 1.08)	**<.001**	1.11 (1.09 ~ 1.13)	**<.001**
P05_ge2	1.09 (1.07 ~ 1.10)	**<.001**	1.08 (1.06 ~ 1.10)	**<.001**	1.08 (1.06 ~ 1.10)	**<.001**	1.12 (1.10 ~ 1.14)	**<.001**
P05_ge3	1.09 (1.07 ~ 1.10)	**<.001**	1.08 (1.06 ~ 1.10)	**<.001**	1.08 (1.06 ~ 1.10)	**<.001**	1.12 (1.10 ~ 1.15)	**<.001**
P05_ge4	1.09 (1.07 ~ 1.11)	**<.001**	1.09 (1.07 ~ 1.10)	**<.001**	1.09 (1.07 ~ 1.10)	**<.001**	1.13 (1.11 ~ 1.16)	**<.001**
P025_ge2	1.09 (1.07 ~ 1.12)	**<.001**	1.08 (1.06 ~ 1.11)	**<.001**	1.08 (1.06 ~ 1.11)	**<.001**	1.11 (1.08 ~ 1.14)	**<.001**
P025_ge3	1.10 (1.08 ~ 1.12)	**<.001**	1.09 (1.06 ~ 1.11)	**<.001**	1.09 (1.06 ~ 1.11)	**<.001**	1.12 (1.09 ~ 1.16)	**<.001**
P025_ge4	1.10 (1.07 ~ 1.12)	**<.001**	1.09 (1.06 ~ 1.11)	**<.001**	1.09 (1.06 ~ 1.11)	**<.001**	1.12 (1.09 ~ 1.15)	**<.001**

Model 1: crude model. Model 2: adjusting age, gender, marital status, educational level, residence, smoking and drinking status, and BMI. Model 3: Model 2+additional adjusting comorbidities (diabetes, cancer, heart disease, stroke and disability). Model 4: Model 3+additional adjusting air pollutants. Abbreviation: HR hazard ratio, CI confidence interval

Table S4 False discovery rate (FDR) adjusted q-values for subgroup interaction tests across nine cold spell definitions.

Subgroups	p075_ge2	p075_ge3	p075_ge4	p05_ge2	p05_ge3	p05_ge4	p025_ge2	p025_ge3	p025_ge4
Age	0.2478	0.2488	0.2469	0.2028	0.1816	0.1821	0.1242	0.1223	0.1208
Education	<0.001	<0.001	<0.001	<0.001	<0.001	<0.001	<0.001	<0.001	<0.001
Gender	0.1207	0.1251	0.1252	0.1407	0.1463	0.1517	0.1782	0.1797	0.1840
Material	0.1570	0.1789	0.1692	0.1239	0.1131	0.0940	0.0572	0.0585	0.0435
BMI	0.2498	0.2497	0.2480	0.1350	0.1246	0.1237	0.0867	0.0743	0.0817
Residence	<0.001	<0.001	<0.001	<0.001	<0.001	<0.001	<0.001	<0.001	<0.001
Alcohol drinking status	0.0303	0.0310	0.0301	0.0293	0.0266	0.0281	0.0573	0.0621	0.0711
Smoking status	0.0323	0.0293	0.0312	0.0558	0.0609	0.0655	0.1207	0.1238	0.1247
Diabetes	0.5604	0.6383	0.6469	0.8894	0.9020	0.9107	1.0006	0.9668	0.9172
Cancer	0.0418	0.0277	0.0270	0.0263	0.0284	0.0254	0.0185	0.0223	0.0176
Heart disease	0.3120	0.2567	0.2464	0.1240	0.1170	0.0928	0.1051	0.0940	0.09148
Stroke	0.8937	0.2567	0.8995	0.9899	0.9930	0.9546	0.8963	0.8944	0.8713
Disability	0.0285	0.0303	0.0302	0.0284	0.0263	0.0270	0.1250	0.1232	0.1233

FDR, false discovery rate.q-values were calculated using the Benjamini-Hochberg procedure across all 117 interaction tests (13 subgroups × 9 cold spell definitions). $q < 0.05$ was considered statistically significant. Values shown are adjusted q-values rounded to four decimal places; $q < 0.001$ are denoted as “<0.001”.

Table S5 Sensitivity analysis of the effect of cold spells on CLD risk after z-score standardization of continuous variables.

Variables	Model1		Model2		Model3		Model4	
	HR (95%CI)	*P*	HR (95%CI)	*P*	HR (95%CI)	*P*	HR (95%CI)	*P*
P075_ge2	2.37 (2.02 ~ 2.78)	**<.001**	2.26 (1.92 ~ 2.67)	**<.001**	2.26 (1.92 ~ 2.67)	**<.001**	4.15 (3.39 ~ 5.08)	**<.001**
P075_ge3	2.28 (1.93 ~ 2.68)	**<.001**	2.17 (1.83 ~ 2.57)	**<.001**	2.17 (1.83 ~ 2.57)	**<.001**	3.77 (3.06 ~ 4.63)	**<.001**
P075_ge4	2.32 (1.97 ~ 2.73)	**<.001**	2.20 (1.86 ~ 2.61)	**<.001**	2.20 (1.86 ~ 2.61)	**<.001**	3.77 (3.06 ~ 4.64)	**<.001**
P05_ge2	2.61 (2.21 ~ 3.08)	**<.001**	2.46 (2.07 ~ 2.92)	**<.001**	2.46 (2.07 ~ 2.92)	**<.001**	4.01 (3.21 ~ 5.01)	**<.001**
P05_ge3	2.64 (2.23 ~ 3.12)	**<.001**	2.48 (2.08 ~ 2.96)	**<.001**	2.48 (2.08 ~ 2.96)	**<.001**	4.07 (3.25 ~ 5.11)	**<.001**
P05_ge4	2.75 (2.31 ~ 3.26)	**<.001**	2.59 (2.16 ~ 3.09)	**<.001**	2.59 (2.16 ~ 3.09)	**<.001**	4.45 (3.52 ~ 5.63)	**<.001**
P025_ge2	2.42 (1.99 ~ 2.94)	**<.001**	2.22 (1.81 ~ 2.72)	**<.001**	2.22 (1.81 ~ 2.72)	**<.001**	3.22 (2.44 ~ 4.25)	**<.001**
P025_ge3	2.48 (2.04 ~ 3.01)	**<.001**	2.28 (1.86 ~ 2.79)	**<.001**	2.28 (1.86 ~ 2.79)	**<.001**	3.42 (2.60 ~ 4.51)	**<.001**
P025_ge4	2.45 (2.01 ~ 2.99)	**<.001**	2.25 (1.83 ~ 2.77)	**<.001**	2.25 (1.83 ~ 2.77)	**<.001**	3.30 (2.49 ~ 4.38)	**<.001**

Model 1: crude model. Model 2: adjusting age, gender, marital status, educational level, residence, smoking and drinking status, and BMI. Model 3: Model 2+additional adjusting comorbidities (diabetes, cancer, heart disease, stroke and disability). Model 4: Model 3+additional adjusting air pollutants. Abbreviation: HR: Hazard Ratio, CI: Confidence Interval

Table S6. Sensitivity analysis of the effect of cold spells on CLD risk after shortening the follow-up period to Wave 3.

Variables	Model 1		Model 2		Model 3		Model 4	
	HR (95%CI)	*P*	HR (95%CI)	*P*	HR (95%CI)	*P*	HR (95%CI)	*P*
P075_ge2	1.08 (1.07 ~ 1.10)	**<.001**	1.07 (1.05 ~ 1.09)	**<.001**	1.07 (1.05 ~ 1.09)	**<.001**	1.12 (1.10 ~ 1.14)	**<.001**
P075_ge3	1.09 (1.07 ~ 1.11)	**<.001**	1.07 (1.05 ~ 1.09)	**<.001**	1.07 (1.05 ~ 1.09)	**<.001**	1.12 (1.10 ~ 1.15)	**<.001**
P075_ge4	1.09 (1.07 ~ 1.11)	**<.001**	1.07 (1.05 ~ 1.09)	**<.001**	1.07 (1.05 ~ 1.09)	**<.001**	1.13 (1.10 ~ 1.15)	**<.001**
P05_ge2	1.11 (1.09 ~ 1.13)	**<.001**	1.09 (1.07 ~ 1.11)	**<.001**	1.09 (1.07 ~ 1.11)	**<.001**	1.14 (1.12 ~ 1.17)	**<.001**
P05_ge3	1.11 (1.09 ~ 1.14)	**<.001**	1.09 (1.07 ~ 1.12)	**<.001**	1.09 (1.07 ~ 1.12)	**<.001**	1.15 (1.12 ~ 1.18)	**<.001**
P05_ge4	1.12 (1.09 ~ 1.14)	**<.001**	1.09 (1.07 ~ 1.12)	**<.001**	1.09 (1.07 ~ 1.12)	**<.001**	1.15 (1.12 ~ 1.18)	**<.001**
P025_ge2	1.17 (1.14 ~ 1.20)	**<.001**	1.14 (1.11 ~ 1.18)	**<.001**	1.14 (1.11 ~ 1.18)	**<.001**	1.20 (1.16 ~ 1.25)	**<.001**
P025_ge3	1.18 (1.15 ~ 1.21)	**<.001**	1.15 (1.11 ~ 1.18)	**<.001**	1.15 (1.11 ~ 1.18)	**<.001**	1.22 (1.17 ~ 1.26)	**<.001**
P025_ge4	1.18 (1.15 ~ 1.21)	**<.001**	1.15 (1.11 ~ 1.18)	**<.001**	1.15 (1.11 ~ 1.18)	**<.001**	1.21 (1.17 ~ 1.26)	**<.001**

Model 1: crude model. Model 2: adjusting age, gender, marital status, educational level, residence, smoking and drinking status, and BMI. Model 3: Model 2+additional adjusting comorbidities (diabetes, cancer, heart disease, stroke and disability). Model 4: Model 3+additional adjusting air pollutants. Abbreviation: HR: Hazard Ratio, CI: Confidence Interval

Table S7 Sensitivity analysis of the effect of cold spells on CLD risk after shortening the follow-up period to Wave 4.

Variables	Model 1		Model 2		Model 3		Model 4	
	HR (95%CI)	*P*	HR (95%CI)	*P*	HR (95%CI)	*P*	HR (95%CI)	*P*
P075_ge2	1.10 (1.08 ~ 1.12)	**<.001**	1.09 (1.07 ~ 1.11)	**<.001**	1.09 (1.07 ~ 1.11)	**<.001**	1.18 (1.16 ~ 1.20)	**<.001**
P075_ge3	1.10 (1.09 ~ 1.12)	**<.001**	1.09 (1.08 ~ 1.11)	**<.001**	1.09 (1.08 ~ 1.11)	**<.001**	1.18 (1.16 ~ 1.20)	**<.001**
P075_ge4	1.11 (1.09 ~ 1.12)	**<.001**	1.10 (1.08 ~ 1.11)	**<.001**	1.10 (1.08 ~ 1.11)	**<.001**	1.18 (1.16 ~ 1.21)	**<.001**
P05_ge2	1.15 (1.13 ~ 1.17)	**<.001**	1.14 (1.12 ~ 1.16)	**<.001**	1.14 (1.12 ~ 1.16)	**<.001**	1.22 (1.19 ~ 1.24)	**<.001**
P05_ge3	1.16 (1.14 ~ 1.18)	**<.001**	1.15 (1.12 ~ 1.17)	**<.001**	1.15 (1.12 ~ 1.17)	**<.001**	1.22 (1.20 ~ 1.25)	**<.001**
P05_ge4	1.17 (1.14 ~ 1.19)	**<.001**	1.15 (1.13 ~ 1.18)	**<.001**	1.15 (1.13 ~ 1.18)	**<.001**	1.23 (1.21 ~ 1.26)	**<.001**
P025_ge2	1.27 (1.24 ~ 1.31)	**<.001**	1.26 (1.23 ~ 1.30)	**<.001**	1.26 (1.23 ~ 1.30)	**<.001**	1.30 (1.26 ~ 1.34)	**<.001**
P025_ge3	1.29 (1.26 ~ 1.32)	**<.001**	1.28 (1.24 ~ 1.31)	**<.001**	1.28 (1.24 ~ 1.31)	**<.001**	1.31 (1.27 ~ 1.35)	**<.001**
P025_ge4	1.29 (1.26 ~ 1.33)	**<.001**	1.28 (1.24 ~ 1.32)	**<.001**	1.28 (1.24 ~ 1.32)	**<.001**	1.31 (1.27 ~ 1.35)	**<.001**

Model 1: crude model. Model 2: adjusting age, gender, marital status, educational level, residence, smoking and drinking status, and BMI. Model 3: Model 2+additional adjusting comorbidities (diabetes, cancer, heart disease, stroke and disability). Model 4: Model 3+additional adjusting air pollutants. Abbreviation: HR: Hazard Ratio, CI: Confidence Interval

Table S8 Sensitivity analysis of the effect of cold spells on CLD risk after excluding participants with diagnosed asthma at baseline.

Variables	Model 1		Model 2		Model 3		Model 4	
	HR (95%CI)	*P*	HR (95%CI)	*P*	HR (95%CI)	*P*	HR (95%CI)	*P*
P075_ge2	1.06 (1.05 ~ 1.08)	**<.001**	1.06 (1.05 ~ 1.07)	**<.001**	1.06 (1.05 ~ 1.07)	**<.001**	1.11 (1.09 ~ 1.12)	**<.001**
P075_ge3	1.06 (1.05 ~ 1.07)	**<.001**	1.06 (1.05 ~ 1.07)	**<.001**	1.06 (1.05 ~ 1.07)	**<.001**	1.10 (1.08 ~ 1.12)	**<.001**
P075_ge4	1.06 (1.05 ~ 1.08)	**<.001**	1.06 (1.05 ~ 1.07)	**<.001**	1.06 (1.05 ~ 1.07)	**<.001**	1.10 (1.08 ~ 1.12)	**<.001**
P05_ge2	1.08 (1.06 ~ 1.09)	**<.001**	1.07 (1.06 ~ 1.09)	**<.001**	1.07 (1.06 ~ 1.09)	**<.001**	1.11 (1.10 ~ 1.13)	**<.001**
P05_ge3	1.08 (1.07 ~ 1.09)	**<.001**	1.07 (1.06 ~ 1.09)	**<.001**	1.07 (1.06 ~ 1.09)	**<.001**	1.12 (1.10 ~ 1.14)	**<.001**
P05_ge4	1.08 (1.07 ~ 1.10)	**<.001**	1.08 (1.06 ~ 1.09)	**<.001**	1.08 (1.06 ~ 1.09)	**<.001**	1.12 (1.10 ~ 1.14)	**<.001**
P025_ge2	1.08 (1.06 ~ 1.10)	**<.001**	1.07 (1.05 ~ 1.09)	**<.001**	1.07 (1.05 ~ 1.09)	**<.001**	1.11 (1.08 ~ 1.14)	**<.001**
P025_ge3	1.09 (1.07 ~ 1.11)	**<.001**	1.08 (1.06 ~ 1.10)	**<.001**	1.08 (1.06 ~ 1.10)	**<.001**	1.12 (1.09 ~ 1.15)	**<.001**
P025_ge4	1.09 (1.07 ~ 1.11)	**<.001**	1.08 (1.06 ~ 1.10)	**<.001**	1.08 (1.06 ~ 1.10)	**<.001**	1.12 (1.09 ~ 1.15)	**<.001**

Model 1: crude model. Model 2: adjusting age, gender, marital status, educational level, residence, smoking and drinking status, and BMI. Model 3: Model 2+additional adjusting comorbidities (diabetes, cancer, heart disease, stroke and disability). Model 4: Model 3+additional adjusting air pollutants. HR: Hazard Ratio, CI: Confidence Interval

Table S9 Sensitivity analysis of the effect of cold spells on CLD risk after excluding participants with diagnosed cancer at baseline.

Variables	Model 1		Model 2		Model 3		Model 4	
	HR (95%CI)	*P*	HR (95%CI)	*P*	HR (95%CI)	*P*	HR (95%CI)	*P*
P075_ge2	1.06 (1.05 ~ 1.07)	**<.001**	1.06 (1.05 ~ 1.07)	**<.001**	1.06 (1.05 ~ 1.07)	**<.001**	1.11 (1.09 ~ 1.12)	**<.001**
P075_ge3	1.06 (1.05 ~ 1.07)	**<.001**	1.06 (1.04 ~ 1.07)	**<.001**	1.06 (1.04 ~ 1.07)	**<.001**	1.10 (1.08 ~ 1.11)	**<.001**
P075_ge4	1.06 (1.05 ~ 1.07)	**<.001**	1.06 (1.04 ~ 1.07)	**<.001**	1.06 (1.04 ~ 1.07)	**<.001**	1.10 (1.08 ~ 1.11)	**<.001**
P05_ge2	1.08 (1.06 ~ 1.09)	**<.001**	1.07 (1.06 ~ 1.08)	**<.001**	1.07 (1.06 ~ 1.08)	**<.001**	1.11 (1.09 ~ 1.13)	**<.001**
P05_ge3	1.08 (1.06 ~ 1.09)	**<.001**	1.07 (1.06 ~ 1.09)	**<.001**	1.07 (1.06 ~ 1.09)	**<.001**	1.11 (1.09 ~ 1.13)	**<.001**
P05_ge4	1.08 (1.07 ~ 1.10)	**<.001**	1.07 (1.06 ~ 1.09)	**<.001**	1.07 (1.06 ~ 1.09)	**<.001**	1.12 (1.10 ~ 1.14)	**<.001**
P025_ge2	1.08 (1.06 ~ 1.10)	**<.001**	1.07 (1.05 ~ 1.09)	**<.001**	1.07 (1.05 ~ 1.09)	**<.001**	1.11 (1.08 ~ 1.14)	**<.001**
P025_ge3	1.09 (1.07 ~ 1.11)	**<.001**	1.08 (1.06 ~ 1.10)	**<.001**	1.08 (1.06 ~ 1.10)	**<.001**	1.12 (1.09 ~ 1.15)	**<.001**
P025_ge4	1.09 (1.07 ~ 1.11)	**<.001**	1.08 (1.06 ~ 1.10)	**<.001**	1.08 (1.06 ~ 1.10)	**<.001**	1.11 (1.09 ~ 1.14)	**<.001**

Model 1: crude model. Model 2: adjusting age, gender, marital status, educational level, residence, smoking and drinking status, and BMI. Model 3: Model 2+additional adjusting comorbidities (diabetes, cancer, heart disease, stroke and disability). Model 4: Model 3+additional adjusting air pollutants. HR: Hazard Ratio, CI: Confidence Interval

Table S10 ROC analysis results of the spatial machine learning model across different cold-spell indicators.

Exposure	Train_Accuracy	Train_AUC	Train_F1	Test_Accuracy	Test_AUC	Test_F1	Test_Precision	Test_Recall	Brier_Score
p075_ge2	0.870714	0.729904	0.234659	0.864692	0.712546	0.16338	0.763158	0.091483	0.111684
p075_ge3	0.870486	0.73069	0.231237	0.863781	0.712825	0.152975	0.75	0.085174	0.11173
p075_ge4	0.870486	0.730501	0.232275	0.866059	0.712924	0.174157	0.794872	0.097792	0.11168
p05_ge2	0.871853	0.729054	0.239351	0.866515	0.710564	0.174648	0.815789	0.097792	0.111706
p05_ge3	0.871512	0.729162	0.236806	0.866515	0.710816	0.174648	0.815789	0.097792	0.111681
p05_ge4	0.87117	0.728519	0.231135	0.866515	0.709993	0.174648	0.815789	0.097792	0.11178
p025_ge2	0.869006	0.725642	0.201389	0.863781	0.708227	0.143266	0.78125	0.078864	0.112648
p025_ge3	0.868778	0.72546	0.197772	0.863781	0.707743	0.148148	0.764706	0.082019	0.112673
p025_ge4	0.868322	0.725293	0.192737	0.863326	0.707839	0.137931	0.774194	0.07571	0.112771

Table S11 Spatial cross-validation performance of nested spatial prediction models across nine cold spell definitions.

Model	GroupKFold_ AUC	GroupKFold_Brier	Rank(M1/M2)
		p075_ge2	
M0	0.5487	0.2188	/
M1	0.5808	0.1300	3
M2	0.5991	0.1298	8
Δ(M2-M1)	+0.0183	-0.0002	/
		p075_ge3	
M0	0.5487	0.2188	/
M1	0.5779	0.1301	4
M2	0.6009	0.1296	6
Δ(M2-M1)	+0.023	-0.0005	/
		p075_ge4	
M0	0.5487	0.2188	/
M1	0.5785	0.1303	4
M2	0.6015	0.1295	6
Δ(M2-M1)	+0.023	-0.0008	/
		p05_ge2	
M0	0.5487	0.2188	/
M1	0.5938	0.1285	2
M2	0.6098	0.1287	9
Δ(M2-M1)	+0.016	+0.0002	/
		p05_ge3	
M0	0.5487	0.2188	/
M1	0.5864	0.1298	1
M2	0.5996	0.1298	9
Δ(M2-M1)	+0.0132	0.0000	/
		p05_ge4	
M0	0.5487	0.2188	/

M1	0.5958	0.1287	1
M2	0.5981	0.1300	9
Δ(M2-M1)	+0.0023	+0.0013	/
		p025_ge2	
M0	0.5487	0.2188	/
M1	0.5570	0.1349	2
M2	0.5820	0.1350	9
Δ(M2-M1)	+0.025	+0.0001	/
		p025_ge3	
M0	0.5487	0.2188	/
M1	0.5604	0.1342	1
M2	0.5825	0.1343	9
Δ(M2-M1)	+0.0221	+0.0001	/
		p025_ge4	
M0	0.5487	0.2188	/
M1	0.5546	0.1356	2
M2	0.5765	0.1359	9
Δ(M2-M1)	+0.0219	+0.0003	/

AUC, area under the receiver operating characteristic curve; Brier, Brier score; GroupKFold, 5-fold cross-validation grouped by city to prevent spatial information leakage. M0: geographic coordinates (longitude and latitude) only; M1: cold spell exposure plus all covariates; M2: full model including exposure, covariates, and coordinates. Δ(M2-M1) denotes the incremental change from M1 to M2. Rank of cold spell exposure was determined by permutation importance within each model. Ranks are shown as M1 rank / M2 rank.

Table S12 Independent test set performance of M1 and M2 models across cold spell definitions.

Exposures	M1_AUC	M2_AUC	ΔAUC(M2-M1)
p075_ge2	0.6841	0.7125	+0.0284
p075_ge3	0.6832	0.7140	+0.0308
p075_ge4	0.6837	0.7135	+0.0298
p05_ge2	0.6864	0.7075	+0.0211
p05_ge3	0.6872	0.7067	+0.0195
p05_ge4	0.6869	0.7040	+0.0171
p025_ge2	0.6753	0.6979	+0.0226
p025_ge3	0.6766	0.6964	+0.0198
p025_ge4	0.6751	0.6958	+0.0207

AUC, area under the receiver operating characteristic curve; M1: cold spell exposure plus all covariates; M2: full model including exposure, covariates, and geographic coordinates. ΔAUC(M2-M1) indicates the incremental improvement in predictive performance attributable to the addition of geographic coordinates. Results were evaluated on a randomly held-out independent test set (20% of the total sample).

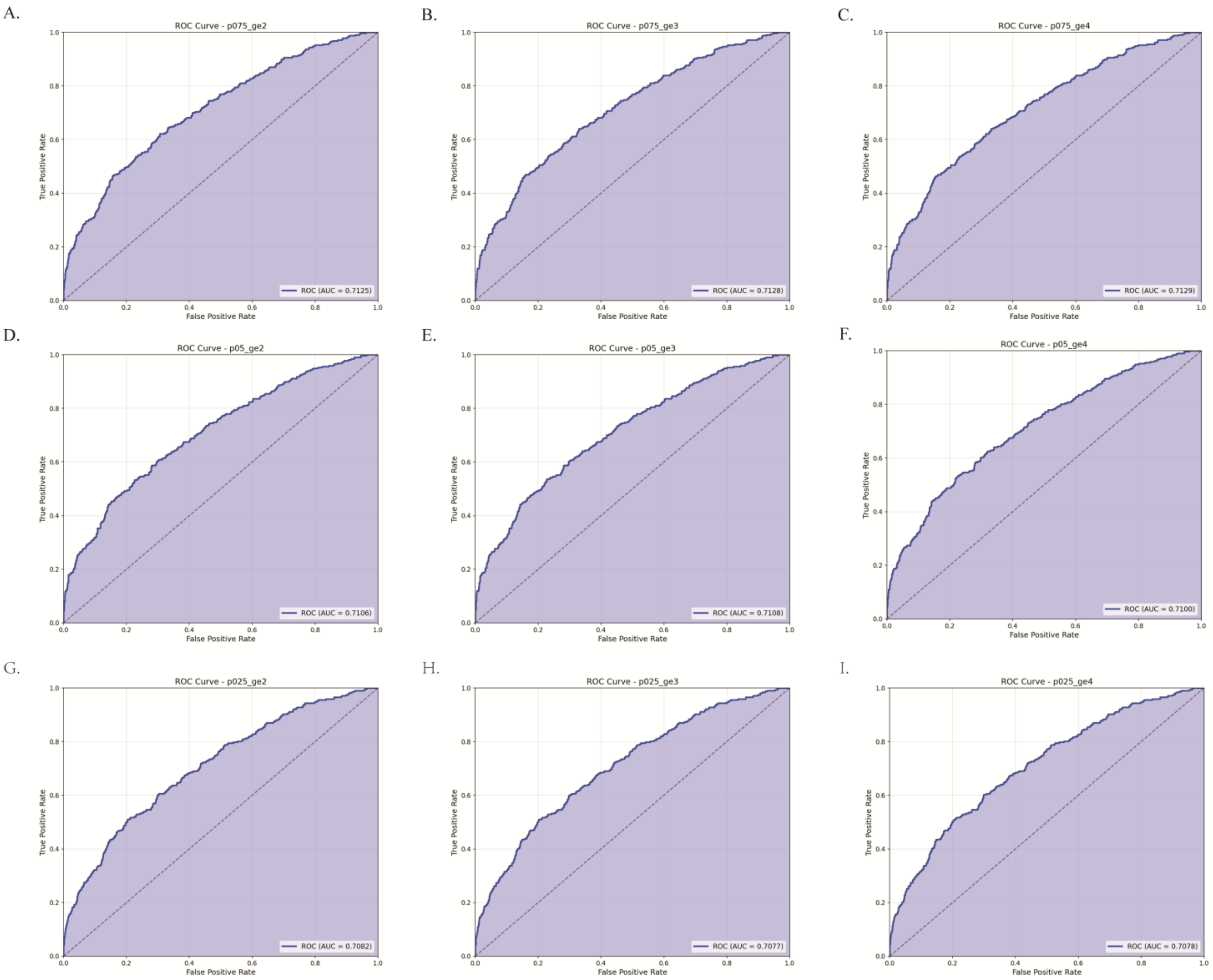

Figure. S1 ROC analysis results of the spatial machine learning model across different cold-spell indicators. A. p075_ge2; B. p075_ge3; C. p075_ge4; D. p05_ge2; E. p05_ge3; F. p05_ge4; G. p025_ge2; H. p025_ge3; I. p025_ge4

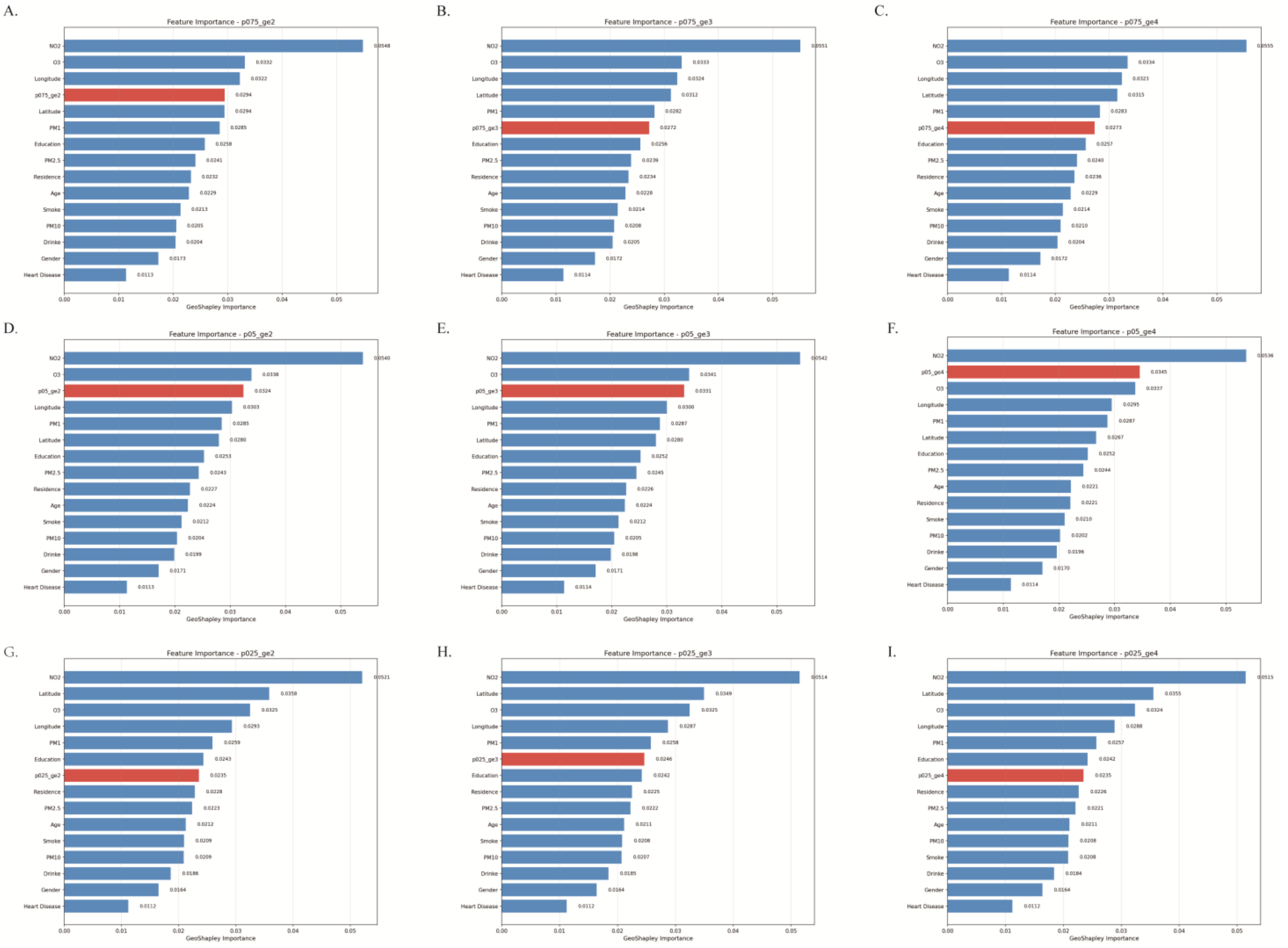

Figure. S2 Feature importance attribution for cold spells using the GeoShapley method in spatial machine learning. A. p075_ge2; B. p075_ge3; C. p075_ge4; D. p05_ge2; E. p05_ge3; F. p05_ge4; G. p025_ge2; H. p025_ge3; I. p025_ge4

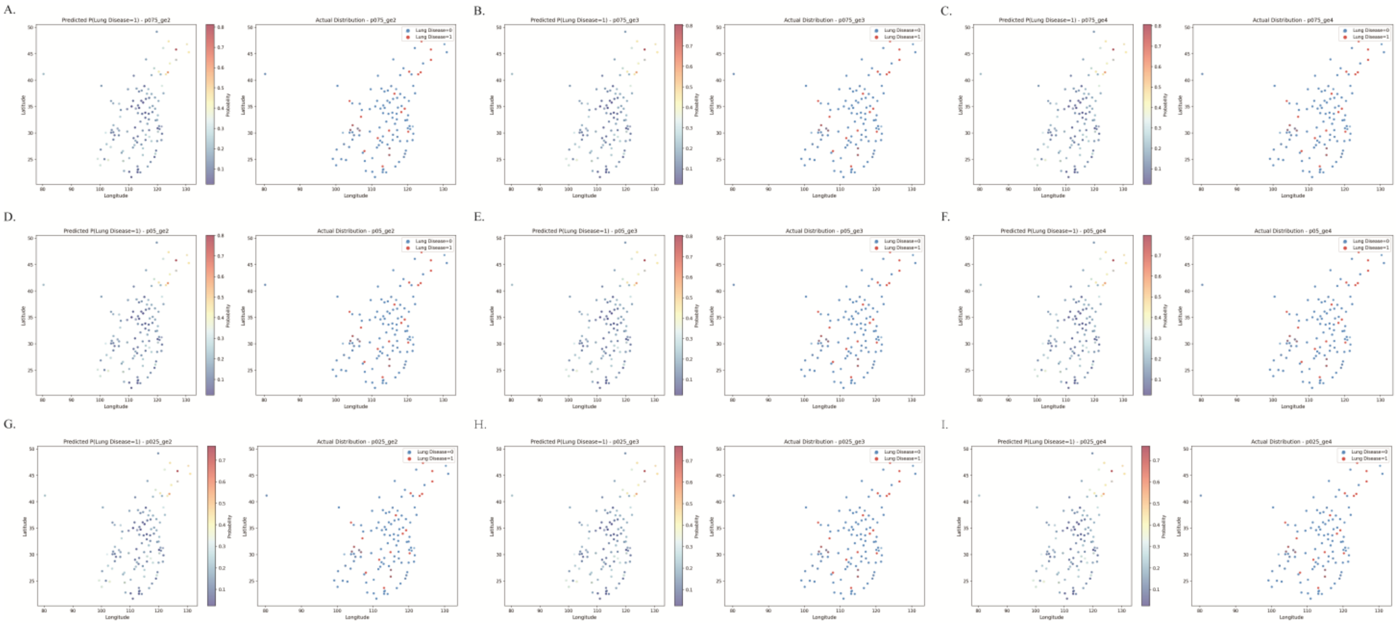

Figure. S3 Cold-Spell Indicators: Spatial maps of the machinel earning model results. A. p075_ge2; B. p075_ge3; C. p075_ge4; D. p05_ge2; E. p05_ge3; F. p05_ge4; G. p025_ge2; H. p025_ge3; I. p025_ge4
